# Serum Glycopatterns as Novel Potential Biomarkers for Diagnosis of Acute-on-Chronic Hepatitis B Liver Failure

**DOI:** 10.1038/srep45957

**Published:** 2017-04-06

**Authors:** Yaogang Zhong, Yonghong Guo, Xiawei Liu, Jiaxu Zhang, Tianran Ma, Jian Shu, Jiajun Yang, Jing Zhang, Zhansheng Jia, Zheng Li

**Affiliations:** 1 Laboratory for Functional Glycomics, College of Life Sciences, Northwest University, Xi’an, P. R. China; 2 Department of infectious diseases, Second Affiliated Hospital of Xi’an Jiaotong University, Xi’an, 710004, P. R. China; 3 Center of infectious diseases, Tangdu Hospital, Fourth Military Medical University, Xi’an, P. R. China

## Abstract

Acute-on-chronic hepatitis B liver failure (ACHBLF) is an increasingly recognized distinct disease entity encompassing an acute deterioration of liver function in patients with cirrhosis, so little is known about the alterations of protein glycopatterns in serum with its development. We aimed to identify the alterations of serum glycopatterns in ACHBLF and probe the possibility of them as novel potential biomarkers for diagnosis of ACHBLF. As a result, there were 18 lectins (e.g., WFA, GSL-II, and PNA) to give significantly alterations of serum glycopatterns in ACHBLF compared with healthy controls (HC) (all *p* ≤ 0.0386). Meanwhile, among these lectins, there were 12 lectins (e.g., WFA, GAL-II, and EEL) also exhibited significantly alterations of serum glycopatterns in ACHBLF compared with HBV-infected chronic hepatitis (cHB) (all *p* ≤ 0.0252). The receiver-operating characteristic (ROC) curve analysis indicated there were 5 lectins (PHA-E + L, BS-I, ECA, ACA, and BPL) had the greatest discriminatory power for distinguishing ACHBLF and HC or cHB, respectively (all *p* ≤ 0.00136). We provided a new basic insight into serum glycopatterns in ACHBLF and investigated the correlation of alterations in serum glycopatterns as novel potential biomarkers for diagnosis of ACHBLF.

Chronic hepatitis B virus (HBV) infection is a well-established cause of liver-related morbidity and mortality, and it is associated with an increased risk of chronic hepatitis, cirrhosis, and hepatocellular carcinoma (HCC)^1^. Acute-on-chronic liver failure (ACLF) has been defined as acute deterioration of liver function in cirrhotic patients over a period of two to four weeks, and is associated with progressive jaundice, hepatic encephalopathy and/or hepatorenal syndrome, and signs of multiorgan dysfunction^2^. ACLF has been shown to carry poor prognosis, especially over the short-term, with an in-hospital mortality ranging from 50% to 66%^3^,^4^. Patients with HBV may experience a severe acute exacerbation of the disease that progresses into liver failure, which is defined as acute-on-chronic hepatitis B liver failure (ACHBLF)^5^. Patients with ACHBLF had a medical history of cHB, serum total bilirubin (TBIL) levels >5 times the upper limit of normal (ULN), and prothrombin activity (PTA) <40%. Antiviral drugs may help reduce the high morbidity and mortality in such patients, especially in places where liver transplantation is not available in these patients^6^. In addition, many patients with ACHBLF die despite significant reduction of HBV DNA, although antiviral drugs provide some short-term survival benefits^7^,^8^. Better diagnostic methods and biomarkers are urgently needed to improve prognosis of patients with ACHBLF at an early stage as this will improve therapeutic effect and patient survival rates.

Protein glycosylation is one of the most complex post-translational modifications (PTMs), because of the large number of enzymatic steps involved, with significant effects on protein folding, stability and activity^9^,^10^. Glycosylation has important biological roles: it takes part in transport of proteins, immune response, communication between cells, oncogenesis, tumor progression. Several glycosylations (e.g., sialylation, fucosylation, and glycosaminoglycans (GAGs)) of human plasma glycoproteins have been proved to be biomarkers for various diseases^11^. Glycans have essential roles in biology and the etiology of many diseases^12^,^13^. Certain types of glycan structures are epitope markers for cancer progression, such as sialyl Lewis^X^, and sialyl Lewis^A^ (carbohydrate antigen 19-9, CA19-9). Lectins have long been used to characterize cell-surface glycans because of their substantial selectivity^14^,^15^. Several lectins, such as ConA, LCA, LTL, PHA-E and PHA-L, are generally used to study altered glycan structures in chronic liver diseases^16^. The advent of high-throughput glycomic techniques enabled the lectin microarrays to observe multiple, distinct binding interactions simultaneously, which have become a primary method to investigate glycosylation of entire samples^15^,^17^.

The aim of the current study was to investigate the correlation of alterations in serum glycosylation related to ACHBLF, and systematically compare different or similar alterations of serum glycopatterns between healthy controls (HC), HBV-infected chronic hepatitis (cHB), and ACHBLF, as well as assess the distribution and localization of specific glycosidic residues in HC and ACHBLF tissues by fluorescence-based lectin histochemistry, and probe the possibility of serum glycopatterns as novel potential biomarkers for diagnosis of ACHBLF.

## Results

### Alterations of serum glycopatterns from male and female subjects of ACHBLF

**Figure 1:**
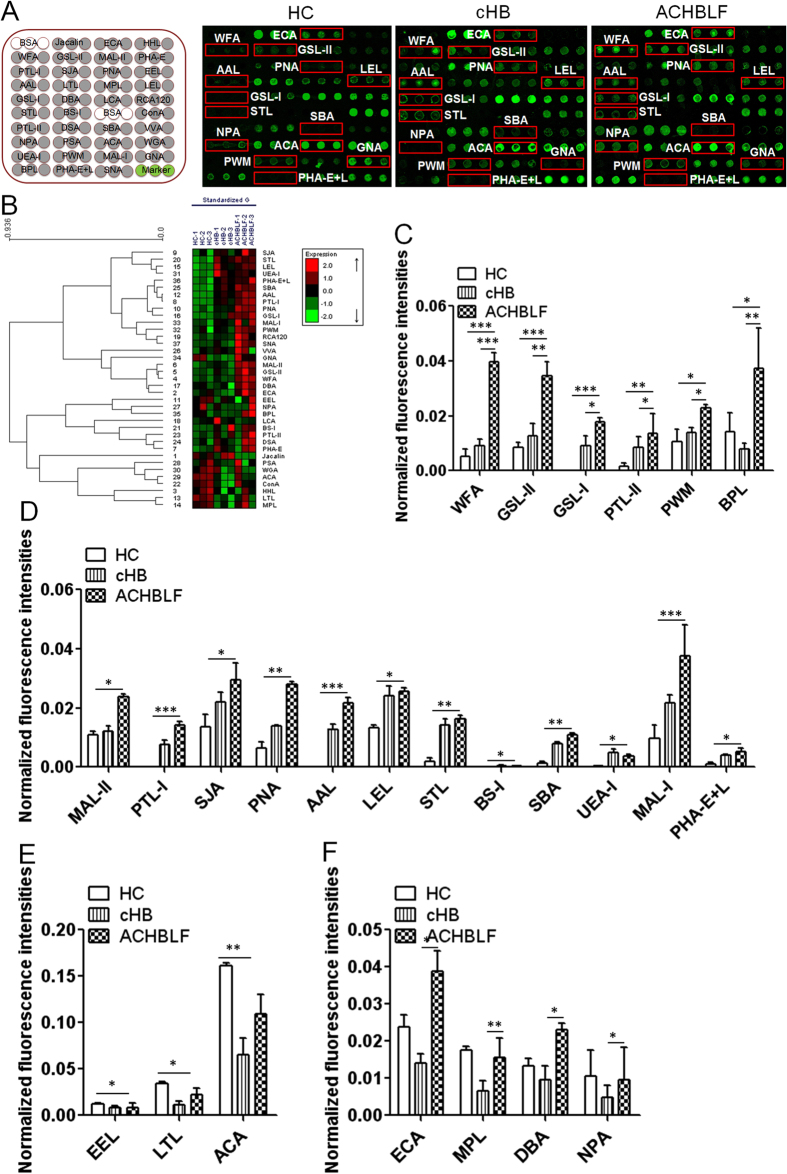
The glycopatterns of serum glycoproteins from male and female HC, cHB, and ACHBLF using the lectin microarrays. (**A**) The layout of the lectin microarray, and the binding profiles of Cy3-labeled sera proteins from HC and patients with cHB, and ACHBLF bound to the lectin microarrays. Lectins showing the difference of glycan expression levels between HC, cHB, and ACHBLF were marked with red frames. (**B**) Heat map and hierarchical clustering of the 37 lectins in three biological replicates. Red and green indicated up- and down-regulated glycans in the ACHBLF (ACHBLF1-3), cHB (cHB1-3) and HC (HC1-3), respectively. (**C**–**F**) Lectins revealed significant differences between HC, cHB, and ACHBLF according to one-way ANOVA (*p < 0.05, **p < 0.01, and ***p < 0.001).

Sera from 30 ACHBLF, 30 cHB, and 30HC were included, and to avoid the differences between subjects and tolerate individual variations, the participants were randomly assigned at the oncoming age stage and the proportionality of male and female subjects in each group was in a 1:1 ratio. The layout of the lectin microarrays and glycopatterns of Cy3-labeled pooled serum proteins from each group bound to the lectin microarrays are shown in Fig. 1A. The normalized fluorescent intensities (NFIs) for each lectin in the three groups are summarized as the mean values ± standard deviations (SD) in Supplementary Table S1. The results showed that there were 25 lectins to give positive signal in pooled sera from the three groups. The generated data from three biological replicates were imported into EXPANDER 6.0 to perform a hierarchical clustering analysis (Fig. 1B). Lectin signal patterns were classified into three categories to evaluate whether the glycopatterns of the sera glycoproteins were altered in ACHBLF: (1) results showing significant increases in NFIs (fold change ≥2, *p* < 0.05), (2) results showing significant decreases in NFIs (fold change ≤0.5, *p* < 0.05), and (3) results showing almost even level in NFIs (fold change range from 0.5 to 2, no significant difference). All based on fold change in pairs (with *p*-values lower than 0.05) with the NFIs of each lectin from HC, cHB, and ACHBLF are showed in Supplementary Table S1. The results showed the terminating with GalNAcα/β1-3/6 Gal binder WFA, the GlcNAc and αGal binder GSL-II and GSL-I, the Gal binder PTL-II, the branched (LacNAc)_n_ binder PWM, and the Galβ1-3GalNAc, the terminal with GalNAc binder BPL, exhibited significantly increased NFIs (all fold change ≥2, *p* = 0.005) in ACHBLF compared with HC and cHB (Fig. 1C).

There were 12 lectins (e.g., MAL-II, PTL-I, and SJA) to show stronger signal with the fold change greater than 2 in ACHBLF compared with HC (Fig. 1D). Among the total, the α-Gal, α-GalNAc, Galα1-3 Gal, and Galα-1-6Glc binder BS-I, the Fucα1-2Galβ1-4Glc(NAc) binder UEA-I exhibited decreased NFIs (all fold change ≤0.44, *p* < 0.05) in ACHBLF compared with cHB. Meanwhile, the Galα1-3(Fucα1-2)Gal binder EEL, Fucα1-2Galβ1-4GlcNAc and Fucα1-3(Galβ1-4)GlcNAc binder LTL, and T antigen binder ACA showed weak signal with the fold change lower than 0.5 in ACHBLF compared with HC (Fig. 1E). However, the Galβ1-4GlcNAc (type II), Galβ1-3GlcNAc (type I) binder ECA, the Galβ1-3GalNAc, GalNAc binder MPL, the αGalNAc, Tn antigen, GalNAcα1-3(Fucα1-2)Gal binder DBA, and the high-Mannose, Manα1-6Man binder NPA was associated with significantly increased NFIs in ACHBLF compared with cHB (all fold change ≥2.33, *p* ≤ 0.005) (Fig. 1F).

### Assessment of serum glycopatterns as potential biomarkers for diagnosis of ACHBLF

**Figure 2:**
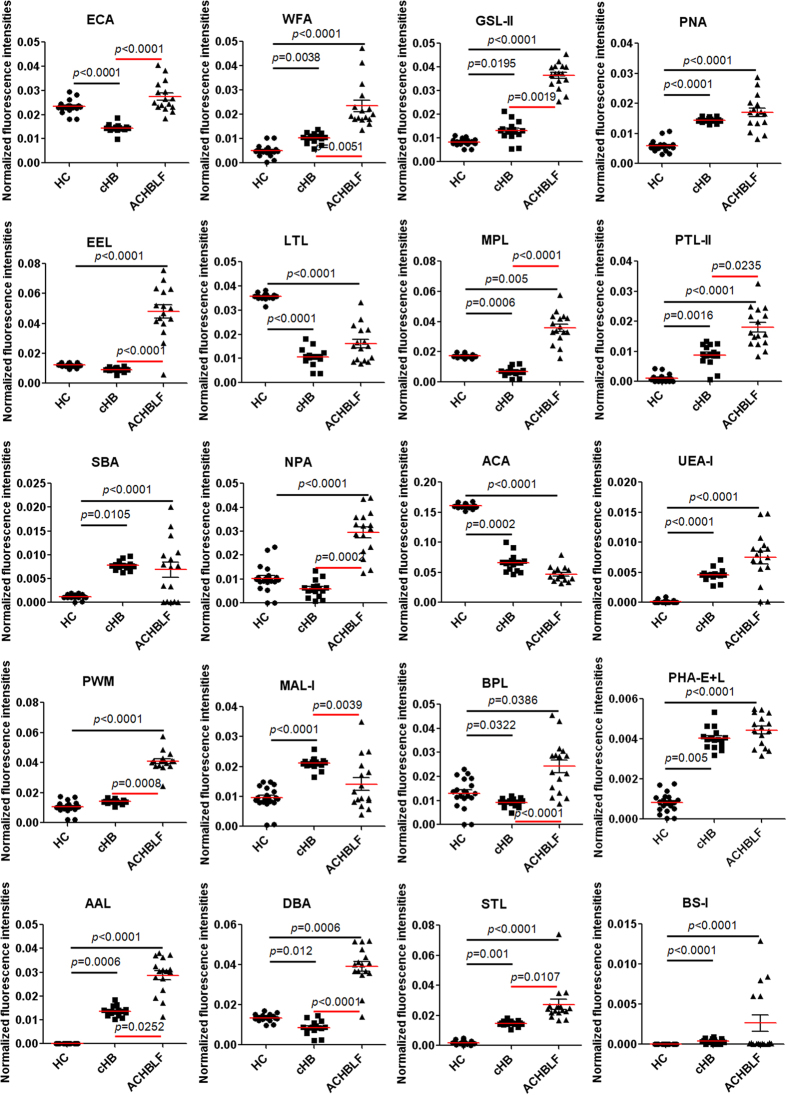
Scatterplots of the data obtained with the 25 candidate lectins against HC score. Red horizontal lines represent the median. Correlation of the data with the progression of ACHBLF was evaluated as significant differences in the medians relative to HC scores by a nonparametric method, the Kruskal–Wallis one-way ANOVA.

**Figure 3:**
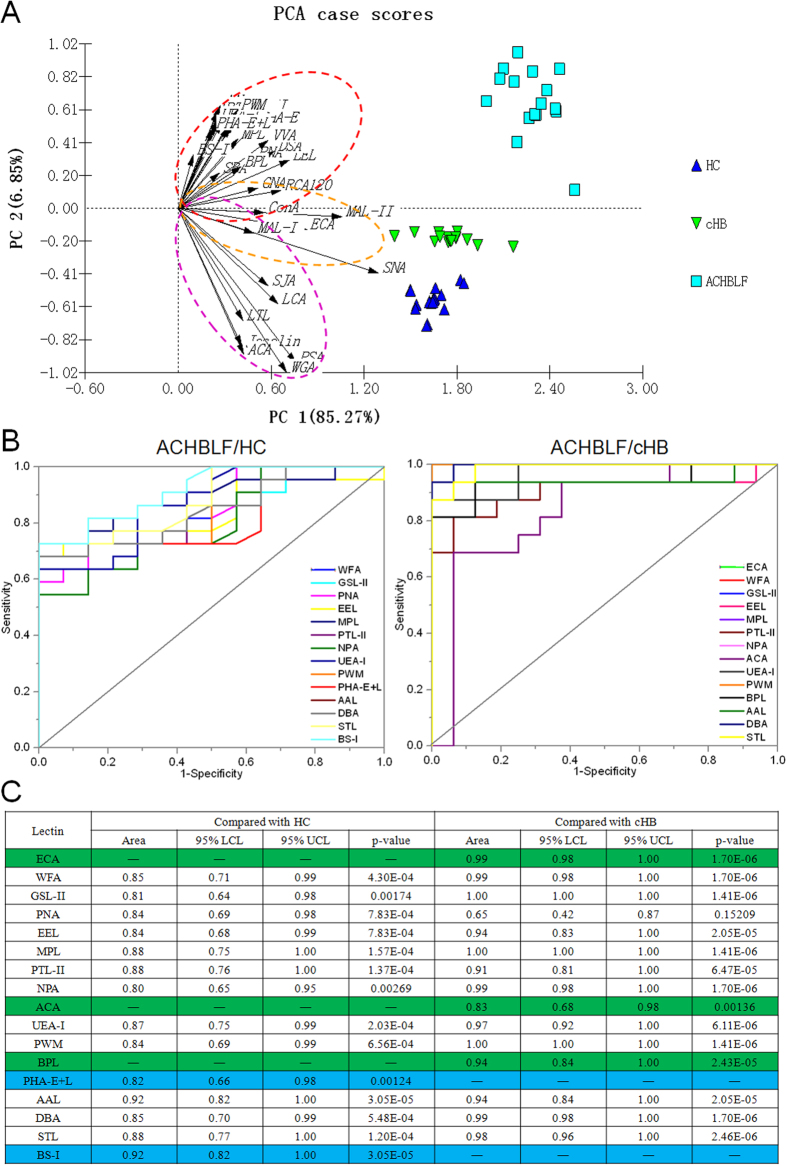
The diagnosis accuracy of the candidate lectins analyzed by PCA, and ROC curve analysis, respectively. (**A**) The normalized glycopattern abundances responses to 3 pools were visualized by PCA. HC, cHB, and ACHBLF were indicated by a blue dotted line, green dotted line, indigo dotted line, respectively. (**B**) AUC of the 17 candidate lectins for HC, cHB, and ACHBLF. (**C**) The detailed information of the selected lectins analyzed by ROC analysis. —: Lectins with an AUC value lower than 0.80.

Based on 25 above candidate lectins (e.g., ECA, WFA, and GSL-II) that exhibited significantly alterations of protein glycopatterns in serum with ACHBLF, and to assess the serum glycopatterns as potential biomarkers for diagnosis of ACHBLF, serum samples of another cohort of ACHBLF (n = 16), cHB (n = 16), and HC (n = 20) were tested using the lectin microarrays independently. There were 18 lectins (e.g., WFA, GSL-II, and PNA) to give significantly alterations of serum glycopatterns in ACHBLF compared with HC (all *p* ≤ 0.0386). Meanwhile, among these lectins, there were 12 lectins (e.g., WFA, GAL-II, and EEL) also exhibited significantly alterations of serum glycopatterns in ACHBLF compared with cHB (all *p* ≤ 0.0252). Notably, ECA showed only significantly up-regulation of serum glycopatterns (*p* < 0.0001), however, MAL-I only showed significantly down-regulation (*p* = 0.0039) of serum glycopatterns in ACHBLF compared with cHB (Fig. 2). The principal component analysis (PCA) was used to provide graphical representations of the relationships between ACHBLF, cHB, and HC, which was generated based on the data of each lectin response patterns together for 52 serum samples. The PCA results showed that the subjects assigned to scatterplots tended to cluster separately to form ACHBLF, cHB, and HC pools with different symbols for each pool in Fig. 3A. Interestingly, it was seen that there were no overlapping area among them, indicating that it was possible to distinguish between ACHBLF, cHB, and HC based on precise alterations in serum glycopatterns.

### Diagnostic accuracy for ACHBLF compared with HC/cHB

Receiver-Operating Characteristic (ROC) analysis was performed to show the diagnosis accuracy of the candidate lectins (Fig. 3B). Lectins with an AUC value greater than 0.80 were selected further for individual candidate analysis. The results indicated there were 17 lectins (e.g., ECA, WFA, and GSL-II) achieved a better diagnosis power for the detection of ACHBLF compared with HC and/or cHB. Among these lectins, there were 12 lectins (e.g., WFA, GSL-II, and PNA) had the discriminatory power for distinguishing ACHBLF and HC/cHB, simultaneously. For example, the AUC curve for WFA was 0.85 (95% confidence interval [CI]: 0.71–0.99; *p* < *0.001*) for the diagnosis of ACHBLF compared with HC, and 0.99 (95% CI: 0.98–1.00; *p* < 0.001) for the diagnosis of ACHBLF compared with cHB, respectively. Notably, the other 5 lectins (PHA-E + L, BS-I, ECA, ACA, and BPL) had the greatest discriminatory power for distinguishing ACHBLF and HC or cHB, respectively. The AUC curve for PHA-E + L and BS-I marked with blue was 0.82 (95% CI: 0.66–0.98; *p* = 0.00124) and 0.92 (95% CI: 0.82–1.00; *p* < 0.001), respectively, which achieved a higher diagnostic accuracy and specificity for the diagnosis of ACHBLF compared with HC. While, the AUC curves for ECA, ACA, and BPL marked with green was 0.99 (95% CI: 0.98–1.00; *p* < 0.001), 0.83 (95% CI: 0.68–0.98; *p* = 0.00136), and 0.94 (95% CI: 0.84–1.00; *p* < 0.001), respectively, which achieved a higher diagnostic accuracy and specificity for the diagnosis of ACHBLF compared with cHB (Fig. 3C).

### Validation of the differential serum glycopatterns

**Figure 4:**
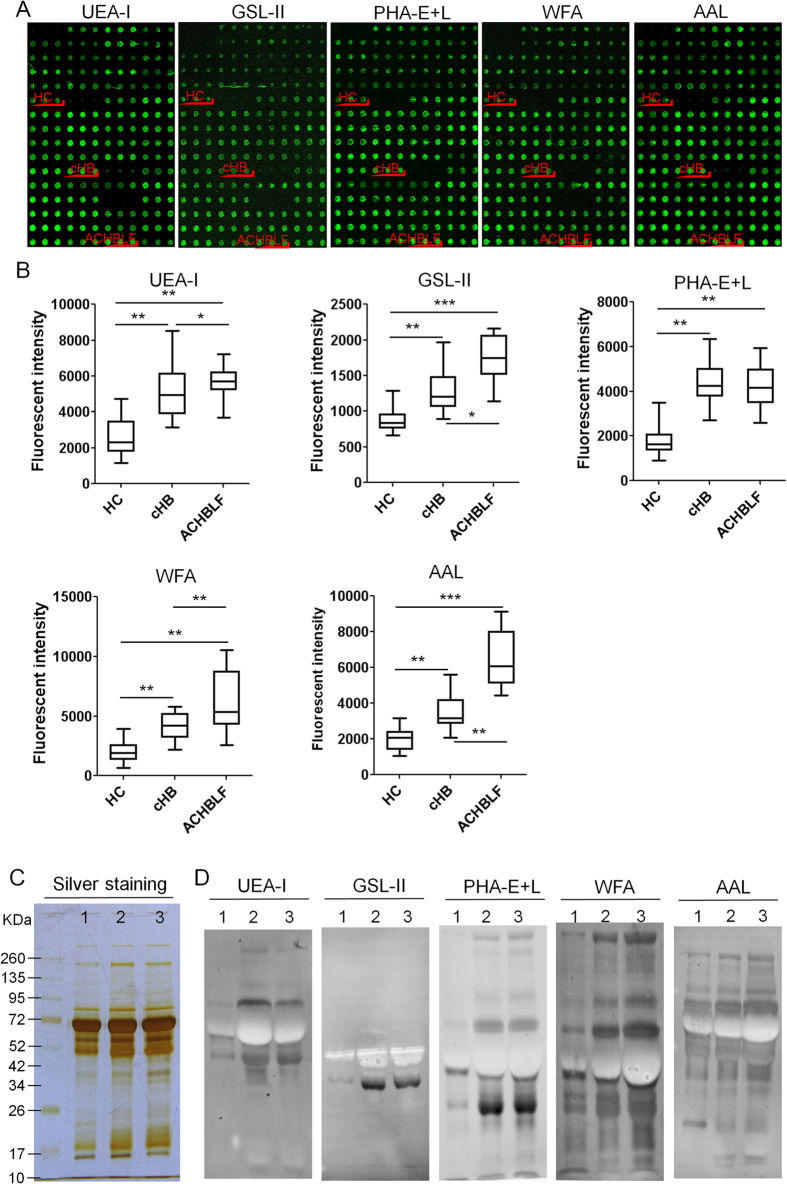
Validation of the differential expression levels of the glycopatterns in the sera associated with the ACHBLF. (**A**) The image of the serum microarrays, which included a total of 60 serum samples ranging from HC (n = 20) and patients with cHB (n = 20) and ACHBLF (n = 20). (**B**) Box plot analysis of the original data obtained from the serum microarrays. Error bars represent 95% confidence intervals for the means. The statistical significance of the differences between HC, cHB, and ACHBLF was indicated by the *p*-value. (*p < 0.05, **p < 0.01, and ***p < 0.001). (**C**) SDS-PAGE analysis. (**D**) The binding pattern of glycoproteins from serum samples of HC, cHB and ACHBLF were analyzed by using 5 lectins (UEA-I, GSL-II, PHA-E + L, WFA, and AAL).

A serum microarray was made to rapidly test the expression levels of the target glycans in individual serum samples by spotting 60 individual samples (20 of ACHBLF, 20 of cHB, and 20 of HC serum samples selected randomly from each group) in the spotting buffer to a concentration of 1 mg/mL on the surface of an epoxy slide according to our previous publication^18^. The layout of serum microarrays was shown in Supplementary Figure S1. Each serum sample was spotted in triplicate, and the results of the serum microarray were shown in Fig. 4A. Five lectins (UEA-I, GSL-II, PHA-E + L, WFA, and AAL) that revealed significant differences (*p* < 0.05) in serum glycopatterns according to the results of the lectin microarrays were selected to validate the differential expression levels of the targeted glycan structures in individual serum samples. As a result, these lectins staining showed increased fluorescence intensities (FIs) in Fig. 4B (p ≤ 0.045), and UEA-I, GSL-II, WFA, and AAL had an increased FIs in ACHBLF compared with cHB (*p* < 0.05), however, PHA-E + L had no significant alteration in ACHBLF compared with cHB (Fig. 4B). These results were generally consistent with the results from the lectin microarrays.

Lectin blotting analyses were performed using the five lectins (UEA-I, GSL-II, PHA-E + L, WFA, and AAL) to validate the differential expression levels of serum glycopatterns between 20 HC, 20 cHB, and 20 ACHBLF (Samples selected randomly from each group). Silver staining showed similar molecular weights and global abundance for proteins from HC, cHB, and ACHBLF, except for one apparent missing bands with molecular weights (Mr) of 26–17 kDa in HC (Fig. 4C). The results showed that UEA-I, GSL-II, and PHA-E + L had a stronger binding pattern in cHB and ACHBLF compared with HC, however, WFA showed an increasing binding pattern from HC to cHB and ACHBLF, AAL showed an increasing trend between HB and cHB and ACHBLF, notably, some glycoproteins bind to AAL showed high expression levels, while some were down-regulated significantly in ACHBLF compared with HC (Fig. 4D). These results were generally consistent with the results from the lectin microarrays.

### Distribution and localization of glycosidic residues in liver tissue sections

**Figure 5:**
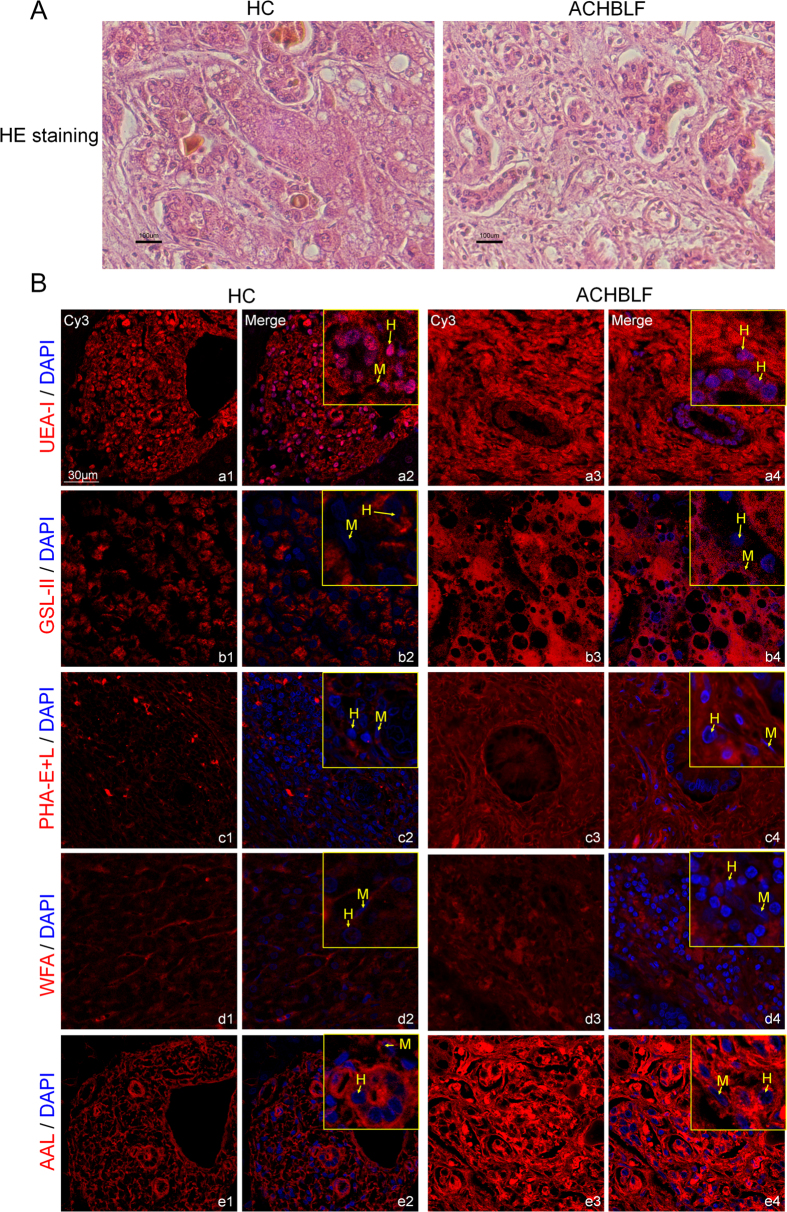
Distribution and localization of glycosidic residues in liver tissue sections. (**A**) Histological examination of the resected liver tissues. (**B**) The images were acquired using the same exposure time and shown on the same scale for the selected lectins (UEA-I (a1-a4), GSL-II (b1-b4), PHA-E + L (c1-c4), WFA (d1-d4), and AAL (e1-e4)) in the Cy3- and DAPI-merge channel. H: Hepatocytes; M: Mesenchymal cells.

To further validate and assess the distribution and localization of specific glycosidic residues in ACHBLF, and tumor adjacent tissue (as HC tissues), the five lectins (UEA-I, GSL-II, PHA-E + L, WFA, and AAL) were selected from candidate lectins showed significant alteration in serum glycopatterns of ACHBLF compared with HC/cHB. Firstly, all the liver tissue samples were histologically examined by hematoxylin and eosin (HE) staining (Fig. 5A). Then, the fluorescence-based lectin histochemistry was performed according to our recent publication^18^. Each Cy3 labeled-lectin detected the targeted sugar structures present on 2 sets of tissue sections HC, and ACHBLF (Supplementary Table S2). The Cy3 staining, Cy3-labeled BSA staining, and monosaccharide inhibition assays for PHA-E + L and WFA were used as the negative controls.

The negative controls showed no positive signal (Supplementary Figure S2), and the selected lectins showed a variety of binding patterns in different regions of pathological liver tissues (Fig. 5, and Table 1). As a result, the expression levels of glycopatterns recognized by the selected lectins increased significantly in high-grade ACHBLF in comparison with HC tissues. UEA-I showed strong binding to the perinuclear region of cytoplasm of hepatocytes and nucleus, and little binding to mesenchymal cells in HC tissues, whereas this binding was clearly increased in the same regions of hepatocytes and was slightly increased in the cytoplasm of mesenchymal cells in ACHBLF tissues (Fig. 5Ba1–a4). GSL-II showed strong binding to the cytoplasm and central cytoplasm of hepatocytes and did not bind to the mesenchymal cells in HC tissues, this binding was clearly increased in the same regions of hepatocytes and was slightly increased in the cytoplasm of mesenchymal cells in ACHBLF tissues (Fig. 5Bb1–b4). PHA-E + L showed strong signals in the membrane and the perinuclear region of the cytoplasm of hepatocytes and even stronger binding to the membrane and the cytoplasm of mesenchymal cells in ACHBLF tissues. However, the signal was lower in the same regions of hepatocytes and mesenchymal cells of HC tissues. Interestingly, PHA-E + L showed scattered particle-like signals in the extracellular matrix of hepatocytes and mesenchymal cells of HC tissues (Fig. 5Bc1–c4). WFA showed strong binding to the cytoplasm, and the perinuclear region of the cytoplasm of hepatocytes and the cytoplasm, the central cytoplasm and the nucleus of mesenchymal cells in ACHBLF tissues, whereas this binding was markedly weakened in both hepatocytes and mesenchymal cells of HC tissues (Fig. 5Bd1–d4). AAL showed strong binding to the membrane and the cytoplasm of hepatocytes and little binding to mesenchymal cells in HC tissues, and this binding was increased in the same region of hepatocytes and mesenchymal cells in ACHBLF tissues (Fig. 5Be1–e4). Those distribution and localization of glycosidic residues in liver tissue sections may provide useful information to find new therapeutic targeting.

## Discussion

Chronic liver diseases are a serious health problem worldwide^19^. The current gold standard to assess structural liver damage is through a liver biopsy which has several disadvantages^20^,^21^. A non-invasive, simple and non-expensive test to diagnose liver pathology would be highly desirable. Protein glycosylation has drawn the attention of many researchers in the search for an objective feature to achieve this goal^22^. Protein glycosylation is the enzymatic addition of sugars or oligosaccharides to proteins. It is the most common form of PTMs of proteins, with as many as 70% of all human proteins estimated to contain one or more glycan chains^23^,^24^, and with 1% of the human genome is involved in glycan production and modification^25^. Although individual liver diseases have their own specific markers, the same modifications seem to continuously reappear in all liver diseases: hyperfucosylation, increased branching and a bisecting *N*-acetylglucosamine^26^. One of the frequently reported glycan alterations in liver disease is a glycan of aberrant fucosylation that is recognized by LCA, which was suggested as a marker for serological monitoring. For example, the LCA-reactive α-fetoprotein (AFP) AFP-L3 is a more specific biomarker for the diagnosis of HCC than the AFP. The latest study observed protein and site specificity of fucosylation in liver-secreted glycoproteins (e.g., serum α1-acid glycoprotein) and *N*-glycan alterations in the development of HCC^27^,^28^,^29^,^30^. With regard to virus-infected chronic hepatitis, the presence of the aberrant glycans in sera is mainly due to glycosyltransferase genes expression changed due to virus genes or proteins regulations. And the other way is through B-lymphocytes autocrine and paracrine stimulation by the receptors of the inflammatory cytokines^31^. Thus lead to the abnormal glycosylation modifications in both the core structures of glycans and the terminal structures. In previous study, the HBV infection has been most extensively investigated in search of changes in glycosylation. Hyperfucosylation, increased branching and the presence of increased bisecting *N*-acetylglucosamine of glycans are clearly associated with different type of liver disease^19^. Gui *et al*. reported that several serum *N*-glycans were altered during the development of liver fibrosis, and higher levels of total agalactosylated biantennary glycans in fibrosis patients with HBV infection than in healthy controls. The biantennary (NA2) and the triantennary (NA3) *N*-glycans decreased significantly with increased severity of fibrosis^32^.

ACHBLF is a devastating syndrome with inordinately high mortality^8^. The short-term prognosis of patients with spontaneous severe acute exacerbation of cHB leading to ACLF-like presentation is extremely poor, with a rarely high mortality rate^32^,^33^,^34^. Liver transplantation has been the only definitive therapy available to salvage this group of patients; however, this is not readily available and feasible in many parts of the world where HBV is highly endemic^34^,^35^. Better diagnostic methods are urgently needed to improve diagnosis of patients with ACHBLF in the short-term for the investigating survival of ACHBLF.

In this study, a lectin microarray was used firstly to investigate sera glycopatterns in the pooled samples and individual sample of subjects, respectively, and systematically compare different or similar alterations of serum glycopatterns between HC, cHB, and ACHBLF. There were 18 lectins (e.g., WFA, GSL-II, and PNA) to give significantly alterations of serum glycopatterns in ACHBLF compared with HC (all *p* ≤ 0.0386). Meanwhile, among these lectins, there were 12 lectins (e.g., WFA, GAL-II, and EEL) also exhibited significantly alterations of serum glycopatterns in ACHBLF compared with cHB (all *p* ≤ 0.0252). The PCA was used to provide graphical representations of the relationships between HC, cHB, and ACHBLF, which showed that the subjects assigned to scatterplots tended to cluster separately to form HC, cHB, and ACHBLF pools that there were no overlapping area, indicating that it was possible to distinguish between HC, cHB, and ACHBLF based on precise alterations in serum glycopatterns. The ROC curve results indicated that there were 17 lectins (e.g., ECA, WFA, and GSL-II) to show high diagnostic accuracy for diagnosis of ACHBLF, among these lectins, 5 lectins (PHA-E + L, BS-I, ECA, ACA, and BPL) had the greatest diagnostic power for distinguishing ACHBLF and HC or cHB, respectively (all *p* < 0.01). Then, a serum microarray was made to rapidly test the expression levels of the target glycans in individual serum samples and validate the differential expression levels of serum glycopatterns between HC, cHB, and ACHBLF based on lectin blotting analyses. These validation results were generally consistent with the results from the lectin microarrays.

Liver tissue is composed mainly of hepatocytes and mesenchymal cells in which glycoproteins are found primarily in the cytoplasmic membrane, central cytoplasm (including the mitochondria and centrosome), the perinuclear region of the cytoplasm (including the endoplasmic reticulum (ER) and Golgi body), and sometimes in the nucleus^36^,^37^,^38^,^39^. Interestingly, a variety of distinct protein glycosylation reactions occur in the ER and Golgi body. In some instances, both the proteins to be glycosylated and the precursor sugar donors must be translocated across the membrane from the cytoplasm to the lumen of the ER^40^,^41^,^42^, and play an important role in many biological process including cell-cell interactions, cell signaling transduction, immune responses, and protein transportation^43^. Likewise, some glycosylated proteins (e.g., CSF1R) are also known to be secreted from the activated macrophages by a PKC-mediated mechanism^39^,^40^,^41^,^42^,^43^,^44^,^45^. Of particular relevance for our study, the fluorescence-based PHA-E + L histochemistry showed stronger signals in the membrane and the perinuclear region of the cytoplasm of hepatocytes and even stronger binding to the membrane and the cytoplasm of mesenchymal cells in ACHBLF tissues. However, the fluorescence-based WFA histochemistry showed a stronger binding to the cytoplasm, and the perinuclear region of cytoplasm of hepatocytes cells, as well as the cytoplasm, the central cytoplasm, and the perinuclear region of cytoplasm of mesenchymal cells in ACHBLF tissues. So, the expression levels of the complex-type *N*-glycans recognized by PHA-E + L and the terminating in GalNAcα/β1-3/6 Gal recognized by WFA in the liver tissue of ACHBLF patients suggested that the secretion of these glycosylated proteins into the serum may be caused by the activation of macrophages, and be associated with hepatic inflammation and cell signaling transduction, the relevant mechanism need to be further studied.

In conclusion, the present study investigated the alterations in serum glycosylation related to ACHBLF in comparison with HC and cHB, and systematically compare different or similar alterations of serum glycopatterns between HC, cHB, and ACHBLF, and probe the possibility of serum glycopatterns as novel potential biomarkers for diagnosis of ACHBLF, as well as assess the distribution and localization of specific glycosidic residues in HC and ACHBLF tissues by fluorescence-based lectin histochemistry, which may provide useful information to find new therapeutic targeting. This study provides insight into the discovery of potential biomarkers for diagnosis of ACHBLF based on the precise alterations of serum glycopatterns.

## Materials and Methods

### Study approval

The collection and use of all human pathology specimens for research presented here were approved by the Ethical Committee of Northwest University, the second affiliated hospital of Xi’an Jiaotong University, and Tangdu Hospital of Fourth Military Medical University, (Xi’an, P. R. China). Written informed consent was received from participants for the collection of their whole serum and tissues. This study was conducted in accordance with the ethical guidelines of the Declaration of Helsinki.

### Serum collection

In this study, a multicenter, randomized, open-label, parallel-group design was used. The collection of human whole blood was performed in accordance with approved guidelines. The collection protocols were provided in the Supplementary Information. Serum samples were collected from 70 HC, and 92 HBV-infected patients (46 cHB and 46 ACHBLF) without antiviral drug treatment from May 2014 through December 2015. Enrollment at each hospital was performed with the use of blocks and randomized for sample balance. The primary clinical and biological data of the patients were summarized in Table 2.

### Lectin microarrays, data acquisition and analysis

To normalize the differences between subjects and to tolerate individual variation, 100 μL of each samples from HC, cHB, and ACHBLF were pooled for lectin microarray detection. The remanent samples from each group were maintained individually for further validation. Total proteins in sera were labeled using Cy3 fluorescent dye and purified using a Sephadex-G25 column^46^,^47^. The detailed information of the lectin microarrays was provided in the Supplementary Information. The acquired images were analyzed at 532 nm for Cy3 detection using Genepix 3.0 software. The average background was subtracted, and values less than the average background ±2 SD were removed from each data point. The median of the effective data points for each lectin was globally normalized to the sum of medians of all effective data points for each lectin in a block. The normalized data of the parallel groups were compared with each other based on fold change, according to the following criteria: fold change ≥2.0 or ≤0.50 (with *p*-values lower than 0.05) in pairs indicated up- or down-regulation, respectively.

### Serum microarrays

A serum microarray was produced by 60 individual serum samples from HC (n = 20) and patients with cHB (n = 20), and ACHBLF (n = 20) according to our previous protocols^48^. Blocking buffer and incubation buffer used in Serum Microarrays could be found in Supplementary Table S3. Detailed information was provided in the Supplementary Information.

### Lectin blotting

Glycoproteins were further analyzed using lectin blotting, which was performed as previously described^47^. Detailed information was provided in the Supplementary information.

### Fluorescence-based lectin histochemistry

The methodology of fluorescence-based lectin histochemistry was performed as previously described^18^. Tissue samples were stained with HE staining^18^,^49^,^50^. To quantitate expression levels of the targeted sugar structures in ACHBLF tissues, five fields of perisinusoidal area were visualized at 80× objective magnification and analyzed with the Image-Pro Plus Version 6.0 software. Detailed information was provided in the Supplementary Information.

### Statistical analysis

The lectin microarray data were expressed as the mean ± SD^51^,^52^. Differences between two arbitrary data sets or multiple data sets were tested using Student’s *t*-test or one-way ANOVA in *SPSS* version 19. The original data were further analyzed by Expander 6.0 (http://acgt.cs.tau.ac.il/expander/), and PCA (Multi-Variate Statistical Package (MVSP), UK) in order to perform hierarchical clustering analysis. ROC curve analysis was carried out to assess classification efficiencies for the diagnosis accuracy of the candidate lectins. Diagnostic accuracy was expressed in terms of area under curve (AUC) values of each lectin between ACHBLF, cHB and HC. P values < 0.05 were considered statistically significant.

## Additional Information

**How to cite this article**: Zhong, Y. *et al*. Serum Glycopatterns as Novel Potential Biomarkers for Diagnosis of Acute-on-Chronic Hepatitis B Liver Failure. *Sci. Rep.* **7**, 45957; doi: 10.1038/srep45957 (2017).

**Publisher's note:** Springer Nature remains neutral with regard to jurisdictional claims in published maps and institutional affiliations.

## Supplementary Material

Supplementary Information

## Figures and Tables

**Table 1:** Lectin-binding pattern in healthy control tissue (HC), and Acute-on-chronic hepatitis B liver failure tissue (ACHBLF) by histochemistry^a^.

Lectin	HC tissue		ACHBLF tissue	
	Hepatocytes	Mesenchymal cells	Hepatocytes	Mesenchymal cells
UEA-I	++^pc+n^	+^pc+n^	+++^pc+n^	++^pc+n+c^
GSL-II	++^c+cc^	+^n^	+++^c+cc^	+^c+cc+n^
PHA-E + L	+^m+pc^	+^m+c^	++^m+pc^	++^m+c^
WFA	+^c+n^	+^cc+pc^	++^c+pc^	+++^c+cc+n^
AAL	++^c+m^	+^c+m^	+++^c+m^	++^c+m^

^a^Binding intensity: +++, very strong binding; ++, strong binding; + , moderate binding; Binding regions: m, cell membrane; c, cytoplasm; cc, central cytoplasm; pc, perinuclear region of cytoplasm; n, nucleus.

**Table 2:** Summarizes the baseline clinical characteristics of study population. [mean ± SEM].

	ACHBLF (n = 46)	cHB (n = 46)	HC (n = 70)
Sex, Female/male	22/24	26/20	36/34
Age (year)	43.82 ± 9.89	39.4 ± 2.57	37.2 ± 2.36
HBsAg/HBeAg (+)	+	+	−
HBV-DNA log10cps/mL	1.27E + 005	3.8E + 006	NA
TBIL (mmol/L)	291.08 ± 34.11	43.23 ± 3.57	13.84 ± 2.21
DBIL (mmol/L)	180.45 ± 23.62	24.3 ± 1.82	NA
IBIL (mmol/L)	95.76 ± 10.98	18.22 ± 3.41	NA
ALT (IU/mL)	495.74 ± 140.75	124.43 ± 23.76	NA
AST (IU/mL)	319.36 ± 40.02	111.39 ± 21.76	NA
TP (g/L)	65.67 ± 3.15	68.69 ± 5.80	NA
ALB (g/L)	34.32 ± 0.89	38.75 ± 5.90	NA
GLB (g/L)	33.56 ± 1.36	29.68 ± 4.87	NA
PTA (%)	31.2 ± 4.46	78.90 ± 9.08	NA
GGT (U/L)	98.42 ± 8.91	67.90 ± 5.67	NA
ALP (IU/L)	145.66 ± 15.86	89.90 ± 9.00	NA
AFP (ng/mL)	198.08 ± 10.87	71.86 ± 7.98	NA

Abbreviations: Data are expressed as mean ± SEM. ALT, alanine aminotransferase; TBIL, total bilirubin; PTA, prothrombin activity; ACHBLF, acute-on-chronic hepatitis B liver failure; cHB, HBV-infected chronic hepatitis; HC, healthy controls; NA, not available.

## References

[b1] Ye Q. H. . Predicting hepatitis B virus-positive metastatic hepatocellular carcinomas using gene expression profiling and supervised machine learning. Nat Med. 9, 416–423 (2003). 12640447 10.1038/nm843

[b2] Jalan R. . Acute-on chronic liver failure. J Hepatol. 57, 1336–1348 (2012). 22750750 10.1016/j.jhep.2012.06.026

[b3] Stadlbauer V. . Effect of extracorporeal liver support by MARS and Prometheus on serum cytokines in acute-on-chronic liver failure. Crit Care. 10, R169 (2006). 17156425 10.1186/cc5119 PMC1794485

[b4] Zheng Y. B. . Development of a sensitive prognostic scoring system for the evaluation of severity of acute-on-chronic hepatitis B liver failure: a retrospective cohort study. Clin Invest Med. 35, 75–85 (2012). 10.25011/cim.v35i2.16291 22469107

[b5] Guo Y. . Upregulated Expression of A20 on Monocytes is Associated With Increased Severity of Acute-on-Chronic Hepatitis B Liver Failure: A Case-Control Study. Medicine (Baltimore). 94, e1501 (2015). 26426612 10.1097/MD.0000000000001501 PMC4616882

[b6] Sen S., Williams R. & Jalan R. The pathophysiological basis of acute-on-chronic liver failure. Liver. 2, 5–13 (2002). 10.1034/j.1600-0676.2002.00001.x 12220296

[b7] Jalan R. & Williams R. Acute-on-chronic liver failure: pathophysiological basis of therapeutic options. Blood Purif. 20, 252–261 (2002). 11867872 10.1159/000047017

[b8] Garg H. . Tenofovir improves the outcome in patients with spontaneous reactivation of hepatitis B presenting as acute-on-chronic liver failure. Hepatology. 53, 774–780 (2011). 21294143 10.1002/hep.24109

[b9] Spiro R. G. Protein glycosylation: nature, distribution, enzymatic formation, and disease implications of glycopeptide bonds. Glycobiology. 12, 43–56 (2002). 10.1093/glycob/12.4.43r 12042244

[b10] Gao X. D. . Alg14 recruits Alg13 to the cytoplasmic face of the endoplasmic reticulum to form a novel bipartite UDP-N-acetylglucosamine transferase required for the second step of N-linked glycosylation. J Biol Chem. 280, 36254–36262 (2005). 16100110 10.1074/jbc.M507569200

[b11] Raynes J. Variations in the relative proportions of microheterogeneous forms of plasma glycoproteins in pregnancy and disease. Biomed Pharmacother. 36, 77–86 (1982). 7126780

[b12] Singh S. . Upregulation of glycans containing 3′ fucose in a subset of pancreatic cancers uncovered using fusion-tagged lectins. J Proteome Res. 14, 2594–2605 (2015). 25938165 10.1021/acs.jproteome.5b00142 PMC4511852

[b13] Song X. . Oxidative release of natural glycans for functional glycomics. Nat Methods. 13, 528–534 (2016). 27135973 10.1038/nmeth.3861 PMC4887297

[b14] Yamashita K. . Lectin microarray technology identifies specific lectins related to lymph node metastasis of advanced gastric cancer. Gastric Cancer. 19, 531–542 (2016). 25840959 10.1007/s10120-015-0491-2

[b15] Hirabayashi J. . Lectin microarrays: concept, principle and applications. Chem Soc Rev. 42, 4443–4458 (2013). 23443201 10.1039/c3cs35419a

[b16] Blomme B. . Alteration of protein glycosylation in liver diseases. J Hepatol. 50, 592–603 (2009). 19157620 10.1016/j.jhep.2008.12.010

[b17] Badr H. A. . Lectin approaches for glycoproteomics in FDA-approved cancer biomarkers. Expert Rev Proteomics. 11, 227–236 (2014). 24611567 10.1586/14789450.2014.897611

[b18] Qin. Y. . Alteration of liver glycopatterns during cirrhosis and tumor progression induced by HBV. Glycoconj J. 3, 125–136 (2016). 10.1007/s10719-015-9645-z 26833199

[b19] Ganne-Carrié N. . Accuracy of liver stiffness measurement for the diagnosis of cirrhosis in patients with chronic liver diseases. Hepatology. 44, 1511–1517 (2006). 17133503 10.1002/hep.21420

[b20] Chen S. H. . Noninvasive assessment of liver fibrosis via spleen stiffness measurement using acoustic radiation force impulse sonoelastography in patients with chronic hepatitis B or C. J Viral Hepat. 19, 654–663 (2012). 22863270 10.1111/j.1365-2893.2012.01588.x

[b21] Shaheen A. A. & Myers R. P. Diagnostic accuracy of the aspartate aminotransferase-to-platelet ratio index for the prediction of hepatitis C-related fibrosis: a systematic review. Hepatology. 46, 912–921 (2007). 17705266 10.1002/hep.21835

[b22] Tsai T. H. . LC-MS profiling of N-Glycans derived from human serum samples for biomarker discovery in hepatocellular carcinoma. J Proteome Res. 13, 4859–4868 (2014). 25077556 10.1021/pr500460k PMC4227556

[b23] Walsh G. & Jefferis R. Post-translational modifications in the context of therapeutic proteins. Nat Biotechnol. 24, 1241–1252 (2006). 17033665 10.1038/nbt1252

[b24] Schirm M. . Identification of unusual bacterial glycosylation by tandem mass spectrometry analyses of intact proteins. Anal Chem. 77, 7774–7782 (2005). 16316188 10.1021/ac051316y

[b25] Hart G. W. & Copeland R. J. Glycomics hits the big time. Cell. 143, 672–676 (2010). 21111227 10.1016/j.cell.2010.11.008 PMC3008369

[b26] Stewart S., Jones D. & Day C. P. Alcoholic liver disease: new insights into mechanisms and preventative strategies. Trends Mol Med. 7, 408–413 (2001). 11530336 10.1016/s1471-4914(01)02096-2

[b27] Oka H. . Multicenter prospective analysis of newly diagnosed hepatocellular carcinoma with respect to the percentage of Lens culinaris agglutinin-reactive alpha-fetoprotein. J Gastroenterol Hepatol. 16, 1378–1383 (2001). 11851836 10.1046/j.1440-1746.2001.02643.x

[b28] Tada T. . Relationship between Lens culinaris agglutinin-reactive alpha-fetoprotein and pathologic features of hepatocellular carcinoma. Liver Int. 25, 848–853 (2005). 15998436 10.1111/j.1478-3231.2005.01111.x

[b29] Durand G. & Seta N. Protein glycosylation and diseases: blood and urinary oligosaccharides as markers for diagnosis and therapeutic monitoring. Clin Chem. 46, 795–805 (2000). 10839767

[b30] Kuno A. . Multilectin assay for detecting fibrosis-specific glyco-alteration by means of lectin microarray. Clin. Chem. 57, 48–56 (2011). 21047982 10.1373/clinchem.2010.151340

[b31] Arnold J. N. . Evaluation of the serum N-linked glycome for the diagnosis of cancer and chronic inflammation. Proteomics. 8, 3284–3293 (2008). 18646009 10.1002/pmic.200800163

[b32] Gui H. L. . Altered serum N-glycomics in chronic hepatitis B patients. Liver Int. 30, 259–267 (2010). 19951379 10.1111/j.1478-3231.2009.02170.x

[b33] Sarin S. K. . Acute-on-chronic liver failure: consensus recommendations of the Asian Pacific Association for the Study of the Liver (APASL). Hepatol Int. 3, 269–282 (2009). 19669378 10.1007/s12072-008-9106-x PMC2712314

[b34] Tsang S. W. . Lamivudine treatment for fulminant hepatic failure due to acute exacerbation of chronic hepatitis B infection. Aliment Pharmacol Ther. 15, 1737–1744 (2001). 11683687 10.1046/j.1365-2036.2001.01107.x

[b35] Chan H. L. . The role of lamivudine and predictors of mortality in severe flare-up of chronic hepatitis B with jaundice. J Viral Hepat. 9, 424–428 (2002). 12431204 10.1046/j.1365-2893.2002.00385.x

[b36] Tsubota A. . Lamivudine monotherapy for spontaneous severe acute exacerbation of chronic hepatitis B. J Gastroenterol Hepatol. 20, 426–432 (2005). 15740488 10.1111/j.1440-1746.2004.03534.x

[b37] Chien R. N., Lin C. H. & Liaw Y. F. The effect of lamivudine therapy in hepatic decompensation during acute exacerbation of chronic hepatitis B. J Hepatol. 38, 322–327 (2003). 12586298 10.1016/s0168-8278(02)00419-1

[b38] George K. Michalopoulos & Marie C. De Frances Liver Regeneration. Science. 276, 60–66 (1997). 9082986 10.1126/science.276.5309.60

[b39] Ohashi K. . Engineering functional two- and three-dimensional liver systems *in vivo* using hepatic tissue sheets. Nat Med. 13, 880–885 (2007). 17572687 10.1038/nm1576

[b40] van Poll D. . Mesenchymal stem cell-derived molecules directly modulate hepatocellular death and regeneration *in vitro* and *in vivo*. Hepatology. 47, 1634–1643 (2008). 18395843 10.1002/hep.22236

[b41] Ocho M. . Application of a glycoproteomics-based biomarker development method: alteration in glycan structure on colony stimulating factor 1 receptor as a possible glycobiomarker candidate for evaluation of liver cirrhosis. J Proteome Res. 13, 1428–1437 (2014). 24422531 10.1021/pr400986t

[b42] Abeijon C. & Hirschberg C. B. Topography of glycosylation reactions in the endoplasmic reticulum. Trends Biochem Sci. 17, 32–36 (1992). 1533966 10.1016/0968-0004(92)90424-8

[b43] Pearse B. R. & Hebert D. N. Lectin Chaperones Help Direct the Maturation of Glycoproteins in the Endoplasmic Reticulum. Biochim Biophys Acta. 1803, 684–693 (2010). 19891995 10.1016/j.bbamcr.2009.10.008 PMC2875293

[b44] Abdulkhalek S. & Szewczuk M. R. Neu1 sialidase and matrix metalloproteinase-9 cross-talk regulates nucleic acid-induced endosomal TOLL-like receptor-7 and -9 activation, cellular signaling and pro-inflammatory responses. Cellular Signalling. 25, 2093–2105 (2013). 23827939 10.1016/j.cellsig.2013.06.010

[b45] Pearce O. M. & Läubli H. Sialic acids in cancer biology and immunity. Glycobiology. 26, 111–128 (2016). 26518624 10.1093/glycob/cwv097

[b46] Zhong Y. . Alteration and localization of glycan-binding proteins in human hepatic stellate cells during liver fibrosis. Proteomics. 15, 3283–3295 (2015). 26058380 10.1002/pmic.201500030

[b47] Qin Y. . Age- and sex-associated differences in the glycopatterns of human salivary glycoproteins and their roles against influenza A virus. J Proteome Res. 12, 2742–2754 (2013). 23590532 10.1021/pr400096w

[b48] Zhong Y. . Identification and localization of xylose-binding proteins as potential biomarkers for liver fibrosis/cirrhosis. Mol Biosyst. 12, 598–605 (2016). 26687723 10.1039/c5mb00703h

[b49] Nie H. . Specific N-glycans of hepatocellular carcinoma cell surface and the abnormal increase of core-α-1,6-fucosylated triantennary glycan via N-acetylglucosaminyltransferases-IVa regulation. Sci Rep. 5, 16007 (2015). 26537865 10.1038/srep16007 PMC4633583

[b50] Zhong Y. . Characterization and sub-cellular localization of GalNAc-binding proteins isolated from human hepatic stellate cells. Biochem Biophys Res Commun. 468, 906–912 (2015). 26616059 10.1016/j.bbrc.2015.11.055

[b51] Zhong Y. . Avian influenza virus infection risk in humans with chronic diseases. Sci Rep. 5, 8971 (2015). 25754427 10.1038/srep08971 PMC4354171

[b52] Qin Y. . Alteration of protein glycosylation in human hepatic stellate cells activated with transforming growth factor-β1. J Proteomics. 75, 4114–4123 (2012). 22659384 10.1016/j.jprot.2012.05.040

