# Supplementary material for: Serum Glycopatterns as Novel Potential Biomarkers for Diagnosis of Acute-on-Chronic Hepatitis B Liver Failure
Source: Sci Rep. 2017 Apr 6;7:45957. doi: 10.1038/srep45957 (PMC5382696; doi:10.1038/srep45957)
Supplement: Supplementary Information [file srep45957-s1.pdf]

Electronic Supplementary Information

Serum Glycopatterns as Novel Potential Biomarkers for Diagnosis of acute-on-chronic hepatitis B liver failure

Yaogang Zhong, Yonghong Guo, Xiawei Liu, Jiaxu Zhang, Tianran Ma, Jian Shu, Jiajun Yang, Jing Zhang, Zhansheng Jia*, Zheng Li*

CONTENTS:

Materials and Methods

Serum collection

Lectin microarrays, data acquisition and analysis

Serum Microarrays

Lectin blotting

Confocal Microscopy

Supplementary Figure S1. Layout of serum microarrays.

Supplementary Figure S2. The negative controls.

Supplementary Table S1. Differential glycopattern of sera from HC, cHB and ACHBLF by the lectin microarray analysis based on data of 25 lectins giving significant signals.

Supplementary Table S2. The information of the liver tissues used for Confocal Microscopy.

Supplementary Table S3. Blocking buffer and incubation buffer used in Serum Microarrays

Materials and Methods

Serum collection

Serum was separated immediately via centrifugation at 1300×g for 10 min at 4 °C. An EDTA-free inhibitor cocktail (Halt protease inhibitor; Thermo Scientific Pierce Protein Research Products, Rockford, IL, USA) was added immediately thereafter at a concentration of 10 μL/mL serum. The produced serum was aliquoted into small portions, immediately rozen on dry ice and stored at -80 °C. The protein concentration was determined using the BCA assay.

Lectin microarrays, data acquisition and analysis

Briefly, 37 lectins with different binding preferences covering *N*- and *O*-linked glycans were spotted on homemade epoxysilane-coated slides. Each lectin was spotted in triplicate per block, with triplicate blocks on one slide. After immobilization, the slides were blocked with blocking buffer containing 2% BSA in 1×PBS (0.01 mol/L phosphate buffer containing 0.15 mol/L NaCl, pH 7.4) for 1 h, rinsed twice with 1× PBST (0.2% Tween 20 in 1× PBS) for 5 min each, and finally rinsed in 1× PBS before drying.

The microarrays were scanned at 70% photomultiplier tube and 100% laser power settings using a Genepix 4000B confocal scanner (Axon Instruments, Foster City, Calif., USA). The acquired images were analyzed at 532 nm for Cy3 detection using Genepix 3.0 software. The average background was subtracted, and values less than the average background ±2 standard deviations (SD) were removed from each data point. The median of the effective data points for each lectin was globally normalized to the sum of medians of all effective data points for each lectin in a block. Each sample was observed consistently on three repeated slides. The normalized medians of each lectin from 9 repeated blocks were averaged, and its SD was counted. The normalized data of the parallel groups were compared with each other based on fold change, according to the following criteria: fold change≥2.0 or ≤0.5 ($p<0.05$) in pairs indicated up-regulation or down-regulation, respectively. Differences between two arbitrary data sets or multiple data sets were tested using Student's t-test or one-way ANOVA in SPSS version 19. The original data were further analyzed using Expander 6.0 (http://acgt.cs.tau.ac.il/expander/) to perform a hierarchical clustering analysis. Receiver-Operating Characteristic (ROC) curve analysis was carried out to assess classification efficiencies for the diagnosis accuracy of the candidate lectins. Diagnostic accuracy was expressed in terms of area under the curve (AUC) values of each lectin between ACHBLF, cHB and HC. P values <0.05 were

considered statistically significant.

Serum Microarrays

In total, 60 individual serum samples from HC (n=20) and patients with cHB (n=20), and ACHBLF (n=20) were dissolved in 1×PBS, pH 7.4, to a concentration of 1 mg/mL before spotting on homemade epoxysilane-coated slides with Stealth micro spotting pins (SMP-10B). After immobilization, the slides were blocked with the blocking and incubation buffers (appendix) for 1 h and rinsed twice with 1× PBS. Cy3-labeled lectin diluted in 0.5 mL of the buffer (Supplementary Table S3) was incubated on the blocked slide for 3 h at room temperature in the dark. The slide was washed and dried via centrifugation at 600 rpm for 5 min. The slide was scanned using a Genepix 4000B confocal scanner at 70% photomultiplier tube and 100% laser power settings, and the acquired images were analyzed at 532 nm for Cy3 detection. All values less than average background ± 2SD were removed from each data point, and the median of the effective data points of each sample was counted. The medians from one group were averaged and expressed as the means ± SD. Differences between two arbitrary groups or multiple groups of medians were tested using Student's t-test or one-way ANOVA for each saliva sample using SPSS version 19.

Lectin blotting

The pooled serum proteins of each group were analyzed by SDS-PAGE, and subsequently, lectin blotting was performed according to the protocol. SDS-PAGE samples were boiled for 4 min at 100°C, mixed with 5×loading buffer and run on a 10% polyacrylamide resolving gel and a 3% stacking gel. Molecular mass standards (Thermo Scientific, Waltham, USA) were run with all gels. Some gels were then stained directly with alkaline silver. For lectin blotting, the proteins in the gels were then transferred to a PVDF membrane (Millipore Corp., USA) with a wet transfer unit (Hoefer Scientific, USA) for 1.5 h at 100 V. After transfer, the membranes were washed twice with TTBS (150 mmol/LNaCl, 10 mmol/LTris-HCl, 0.05% Tween-20, pH 7.5) and then blocked for 1 h with Carbo-Free Blocking Solution (Vector, Burlingame, CA) at room temperature. On the basis of silver staining of the gels after transfer, it was evident that lower-molecular-mass proteins (less than 50 kDa) transferred more thoroughly to the blots. Hence, if any bias was present in the final results, it favored the detection of proteins smaller than 50 kDa during lectin blotting. The membranes were then washed again and

incubated with Cy5-labeled (GE Healthcare, Buckinghamshire, UK) lectins (2 μg/mL in Carbo-Free Blocking Solution) with gentle shaking overnight at 4°C in the dark. The membranes were then washed twice each for 10 min with TTBS and scanned on the red fluorescence channel (635 nm excitation/650 nm LP emission) with a voltage of 800 PMT using a phosphorimager (Storm 840, Molecular Dynamics Inc. USA).

Confocal Microscopy

For confocal microscopy, the formalin-fixed paraffin-embedded tissue sections were dewaxed and hydrated in a series of different concentrations of dimethylbenzene and ethanol. Tissues stained with Cy3-labeled lectins for cytochemistry, then the tissues were blocked with 5% BSA at 37°C for 30 min. Following that, the cells were incubated with the Cy3-labeled lectins diluted at a final concentration of 20-30 μg/mL at 4°C in the dark over night. After that, they were further stained with 1 μg/mL of DAPI (Roche, Basel, CH) for 10 min and a final rinse was performed. Laser scanning confocal microscope FV 1000 (Olympus, Tokyo, JPN) was used to collect the images with the merge channels of Cy3 and/or DAPI. To quantitate expression levels of the targeted sugar structures in ACHBLF tissues, five fields of perisinusoidal area were visualized at 80×objective magnification and analyzed with the Image-Pro Plus Version 6.0 software.

Supplementary Figures

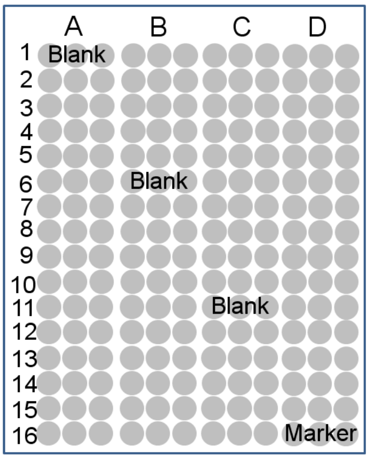

Supplementary Figure S1. Layout of serum microarrays. A1, B6, C11: blank; D16: marker; B1-A6: healthy controls; C6-B11: patients with cHB; D11-C16: patients with ACHBLF.

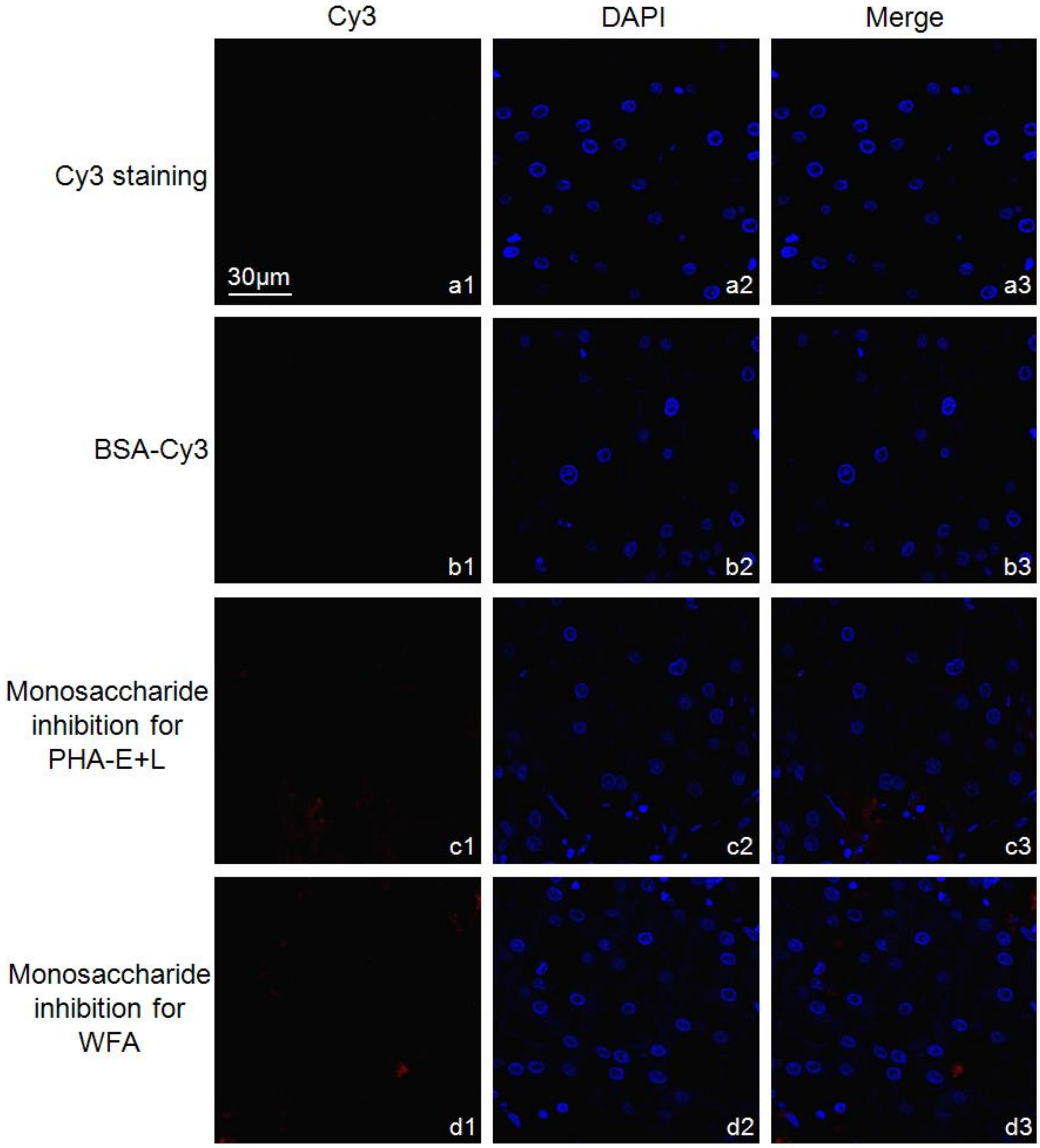

Supplementary Figure S2. Monosaccharide inhibition for lectin histochemical approach in ACHBLF tissues, The images were acquired at the same exposure time and shown on the same scale for each lectin with Cy3 channel, Cy3 and DAPI merge channel, respectively (80×objective magnification). a1-a3: Cy3 Staining; b1-b3: BSA-Cy3 Staining; c1-c3: GlcNAc inhibition (100 mM GlcNAc) for PHA-E+L; d1-d3: GalNAc inhibition (200 mM GalNAc) for WFA.

Supplementary Table S1. Differential glycopattern of sera from HC, cHB and ACHBLF by the lectin microarray analysis based on data of 25 lectins giving significant signals.

Lectins	Specificity	Normalized fluorescent intensity,NFI±SD[a]			Fold change (*p*-value)[b]		
		HC	cHB	ACHBLF	cHB/HC	ACHBLF /HC	ACHBLF /cHB
ECA	Galβ1-4GlcNAc (type II), Galβ1-3GlcNAc (type I)	0.024±0.006	0.0141±0.004	0.038±0.008	—	—	2.72 (0.032)
WFA	terminating in GalNAcα/β1-3/6Gal	0.005±0.005	0.009±0.004	0.041±0.006	—	8.05 (<0.001)	4.61 (<0.001)
GSL-II	GlcNAc and agalactosylated tri/tetra antennary glycans	0.008±0.003	0.013±0.008	0.0381±0.009	—	4.43 (<0.001)	2.98 (0.0089)
MAL-II	Siaα2-3Galβ1-4Glc(NAc)/Glc, Siaα2-3Gal, Siaα2-3, Siaα2-3GalNAc	0.011±0.002	0.012±0.003	0.022±0.004	—	2.04 (0.032)	—
PTL-I	GalNAc, GalNAcα1-3Gal, GalNAcα1-3Galβ1-3/4Glc	/	0.008±0.002	0.015±0.002	***(<0.001)	***(<0.001)	—
SJA	Terminal in GalNAc and Gal, anti-A and anti-B human blood group	0.014±0.007	0.022±0.006	0.035±0.001	—	2.54 (0.048)	—
PNA	Galβ1-3GalNAcα-Ser/Thr(T)	0.006±0.004	0.014±0.006	0.026±0.004	2.17(0.002)	4.12 (0.003)	—
EEL	Galα1-3(Fucα1-2)Gal (blood group B antigen)	0.012±0.002	0.009±0.003	0.006±0.001	—	0.48 (0.012)	—
AAL	Fucα1-6 GlcNAc(core fucose), Fucα1-3(Galβ1-4)GlcNAc	/	0.013±0.003	0.022±0.003	***(<0.001)	***(<0.001)	—
LTL	Fucα1-2Galβ1-4GlcNAc, Fucα1-3(Galβ1-4)GlcNAc, anti-H blood group specificity	0.035±0.003	0.011±0.007	0.014±0.001	0.33(0.017)	0.41 (0.026)	—
MPL	Galβ1-3GalNAc, GalNAc	0.017±0.002	0.006±0.001	0.016±0.007	0.37(0.003)	—	2.47 (0.004)
LEL	(GlcNAc)n, high mannose-type N-glycans	0.013±0.002	0.024±0.006	0.026±0.002	—	2.00 (0.044)	—

GSL-I	αGalNAc, αGal, anti-A and B	/	0.009±0.006	0.018±0.002	***(<0.001)	***(<0.001)	2.00 (0.005)
DBA	αGalNAc, Tn antigen, GalNAcα1-3((Fucα1-2))Gal (blood group A antigen)	0.013±0.003	0.010±0.006	0.022±0.003	—	—	2.33 (0.037)
STL	trimers and tetramers of GlcNAc, core (GlcNAc) of N-glycan, oligosaccharide containing GlcNAc and MurNAc	0.002±0.002	0.014±0.004	0.017±0.002	7.73(<0.001)	9.29 (0.004)	—
BS-I	α-Gal, α-GalNAc, Galα1-3Gal, Galα1-6Glc	/	0.0004±0.0003	0.0002±0.0001	***(<0.001)	***(<0.001)	0.48 (0.048)
PTL-II	Gal, blood group H , T-antigen	0.001±0.002	0.006±0.006	0.015±0.001	4.54(0.002)	10.34 (0.044)	2.28 (0.007)
SBA	α- or β-linked terminal GalNAc, (GalNAc)n, GalNAcα1-3Gal, blood-group A	0.001±0.001	0.008±0.001	0.010±0.002	6.50(<0.001)	8.27 (0.045)	—
NPA	High-Mannose, Manα1-6Man	0.011±0.002	0.005±0.001	0.014±0.001	0.45(<0.001)	—	2.89 (0.012)
ACA	Galβ1-3GalNAcα-Ser/Thr (T antigen), sialyl-T(ST) tissue staining patterns are markedly different than those obtained with either PNA or Jacalin	0.161±0.005	0.065±0.003	0.079±0.005	0.41(0.015)	0.49 (0.004)	—
UEA-I	Fucα1-2Galβ1-4Glc(NAc)	0.0002±0.0001	0.006±0.001	0.003±0.001	24.69(<0.001)	10.85 (0.021)	0.44 (0.036)
PWM	Branched $(LacNAc)_n$	0.010±0.004	0.012±0.003	0.024±0.004	—	2.34 (0.022)	2.06 (0.019)
MAL-I	Galβ1-4GlcNAc, Siaα2-3Gal, Galβ1-3GlcNAc, Siaα2-3	0.010±0.007	0.022±0.005	0.035±0.001	2.25(<0.001)	3.62 (<0.001)	—
BPL	Galβ1-3GalNAc, Terminal GalNAc	0.014±0.001	0.008±0.002	0.043±0.002	—	3.07 (0.011)	5.39 (0.005)
PHA-E+L	Bisecting GlcNAc, bi-antennary N-glycans, tri- and tetra-antennary complex-type N-glycan	0.0009±0.008	0.004±0.001	0.005±0.001	4.24(<0.001)	5.91 (0.047)	—

a Signal intensities obtained for 9 repeated blocks in three repeated slides were normalized and averaged, and the ratios of ACHBLF vs.HC, cHB (ACHBLF /HC AND ACHBLF /cHB) were calculated. HC: healthy control; cHB: HBV-infected patients with chronic liver diseases; ACHBLF: acute-on-chronic hepatitis B liver failure

b /, negative signal; －, no significant difference; *$p<0.05$; **$p<0.01$; ***$p<0.001$.

Supplementary Table S2. The information of the liver tissues used for Confocal Microscopy

No.	Gender	Age	Tissue type	Technique diagnosis
Dlv031304	Male	44	Liver	HBV-induced severe hepatitis
Dlv060331	male	40		

Supplementary Table S3. Blocking buffer and incubation buffer used in Serum Microarrays

Lectin	Blocking buffer & Incubation buffer
WFA	0.1mmol/L Ca^{2+}, 0.5mg/mL BSA in 10mmol/L HEPES buffered saline, pH 8.5
GSL-II	0.1mmol/L Ca^{2+}, 0.5mg/mL BSA in 10mmol/L HEPES buffered saline, pH 8.5
AAL	0.15mol/L Na^{+}, 1 mmol/L fucose and 0.5mg/mL BSA in 10mmol/L HEPES, pH 7.5
UEA-I	0.1mmol/L Ca^{2+}, 0.5mg/mL BSA in 10mmol/L HEPES buffered saline, pH 8.5
PHA-E+L	0.1mmol/L CaCl2, 1mmol/L galactose and 0.5mg/mL BSA in 10mmol/L HEPES, pH 7.5-8.5
